# Healthcare resource utilization and associated cost of patients with bone metastases from solid tumors who are naïve to bone-targeting agents: a comparative analysis of patients with and without skeletal-related events

**DOI:** 10.1007/s10198-020-01247-z

**Published:** 2021-01-18

**Authors:** Fränce Hardtstock, Zeki Kocaata, Thomas Wilke, Axel Dittmar, Marco Ghiani, Vasily Belozeroff, David J. Harrison, Ulf Maywald, Hans Tesch

**Affiliations:** 1 Ingress-Health HWM GmbH, Alter Holzhafen 19, 23966 Wismar, Germany; 2 IPAM e.V., University of Wismar, 23966 Wismar, Germany; 3 Amgen Inc., Thousand Oaks, CA USA; 4 AOK PLUS, Sternplatz 7, 01067 Dresden, Germany; 5 Centrum for Hematology and Oncology, Im Prüfling 17-19, 60389 Frankfurt a. Main, Germany

**Keywords:** Bone metastasis, Skeletal-related events, Healthcare resource utilization, Economic burden, Claims data, Bone-targeting agents, I11

## Abstract

**Background:**

This study analyzes the impact of skeletal-related events (SRE) on healthcare resource utilization (HCRU) and costs incurred by patients with bone metastases (BM) from solid tumors (ST), who are therapy-naïve to bone targeting agents (BTAs).

**Methods:**

German claims data from 01/01/2010 to 30/06/2018 were used to conduct a retrospective comparative cohort analysis of BTA-naive patients with a BM diagnosis and preceding ST diagnosis. HCRU and treatment-related costs were compared in two matched cohorts of patients with and without a history of SREs, defined as pathological fracture, spinal cord compression, surgery to bone and radiation to bone. The first SRE was defined as the patient-individual index date. Conversely, for the non-SRE patients, index dates were assigned randomly.

**Results:**

In total, 45.20% of 9,832 patients reported experiencing at least one SRE (*n* = 4444) while 54.80% experienced none (*n* = 5388); 2,434 pairs of SRE and non-SRE patients were finally matched (mean age: 70.87/71.07 years; females: 39.07%/38.58%). Between SRE and non-SRE cohorts, significant differences in the average number of hospitalization days per patient-year (35.80/30.80) and associated inpatient-care costs (14,199.27€/10,787.31€) were observed. The total cost ratio was 1.16 (*p* < 0.001) with an average cost breakdown of 23,689.54€ and 20,403.27€ per patient-year in SRE and non-SRE patients.

**Conclusion:**

The underutilization of BTAs within a clinical setting poses an ongoing challenge in the real-world treatment of BM patients throughout Germany. Ultimately, the economic burden of treating SREs in patients with BM from ST was found to be considerable, resulting in higher direct healthcare costs and increased utilization of inpatient care facilities.

**Supplementary Information:**

The online version contains supplementary material available at 10.1007/s10198-020-01247-z.

## Introduction

Bone is a very common site for cancer metastases from solid tumors [1, 2]. Although a multitude of malignancies can cause bone metastases (BM), BM are most prevalent in breast and prostate cancers (65–75%) as well as lung cancer (30–40%) [2–4]. BM can result in serious skeletal complications, known as skeletal-related events (SRE) which are commonly defined as pathologic fractures, radiation or surgery to the bone and spinal cord compression [4, 5]. SREs are known to be related to an increased mortality and a decreased health-related quality of life due to severe pain as well as impaired mobility [3]. Further, SRE-associated complications and symptoms are typically treated by palliative radiotherapy or additional surgery leading to negative or even traumatic consequences impacting patients` quality of life [1, 6].

As a preventative measure against SREs, treatment initiation with bone targeting agents (BTA), such as intravenous bisphosphonates [IVBPs, e.g. zoledronic acid (ZA)] or the monoclonal antibody denosumab, is recommended upon confirmation of a BM diagnosis [7–13]. Nevertheless, despite the existing evidence that treatment with either IVBPs or denosumab can delay the occurrence of SREs, prompt and sufficient pharmaceutical management is not necessarily present in clinical practice [2, 7]. Such undertreatment may lead to a substantial patient burden as well as a high healthcare resource utilization (HCRU) and cost [7–14]. While the general burden of SREs has been assessed for different European countries and previous research on the cost-effectiveness of denosumab has been performed [15], HCRU and costs associated with SREs in patients untreated with BTAs are not known so far. Thus, the objective of our analysis was to assess the HCRU and costs in BTA-naïve patients with BM from solid tumors in Germany to investigate the economic consequences of undertreating or delaying treatment of cancer patients at risk of SREs. To estimate the incremental cost of SREs, patients who developed SREs were compared to patients who did not develop SREs after an incident BM diagnosis, by utilizing a high-dimensional propensity score (hdPS) methodology.

## Methods

### Study design and data source

This study was a retrospective comparative cohort analysis of patients with BM from solid tumors who were non-users of BTAs. The anonymized claims dataset that was used to conduct the study was provided by AOK PLUS, a German public sickness fund insuring about 3.2 million people in the eastern region of Germany. The statutory insurance dataset included complete information on all hospitalizations, as well as outpatient consultations including data on all diagnoses, treatments, procedures, prescriptions (Rx) and related costs for the period from 01/01/2010 until 30/06/2018 for all patients with BM. As the dataset was anonymized, no ethical approval was required. Approval from the data owner was provided prior to start of analysis.

### Patient eligibility criteria

We included patients with at least one inpatient or two confirmed outpatient BM diagnoses (ICD-10 code C79.5) in two different quarters between 01/01/2011 and 30/06/2017, without a previous diagnosis of BM in the pre-index period in 2010. No specific criteria related to the time interval between confirmed outpatient codes were used. In addition, at least one inpatient or confirmed outpatient diagnosis of a solid tumor (ST, ICD-10 code C00-C76) within the 12 month period before or 3 month period after the patient-specific date of an incident BM diagnosis was required. Consideration of patients with an ST diagnosis up to 3 months after the first BM diagnosis was permitted to allow for the possibility that patients could be diagnosed with BM first and a primary ST shortly thereafter. Further inclusion criteria included being 18 years of age at the time of incident BM diagnosis and a continuous insurance history with the AOK PLUS sickness fund for 12 months preceding the incident BM diagnosis until the end of data availability (30/06/2018) or death, whichever came first.

All above patients with an incident BM from ST were assigned to two groups: SRE or non-SRE patients, based on whether these patients developed an SRE after the incident BM diagnosis. SREs were observed by documented diagnosis and procedure codes (see Supplementary Table 1) between the incident BM diagnosis date and 30/06/2017 (to allow for a minimum of a 12-month follow-up period after the first observed SRE event). The first SRE after BM date was defined as the patient-individual index date. Conversely, for the non-SRE patients, an index date was assigned randomly, based on the distribution of index dates within the SRE group. In the last step, BTA-naïve patients were selected based on prescription information of denosumab and IVBPs (see Supplementary Table 2). Patients from both groups (SRE/non-SRE) were excluded if they received a BTA therapy (i) before the incident BM diagnosis; or (ii) after incident BM diagnosis but before the date of the first SRE diagnosis (or the randomly assigned index date in the non-SRE group).

### High-dimensional propensity score matching

The hdPS matching methodology was used to compare patients who developed SREs after an incident BM diagnosis following a ST diagnosis, with patients who did not develop SREs. We chose hdPS because it does not simply account for a large number of pre-defined patient and disease characteristics, but also captures unobservable confounding factors that cannot be expressed by pre-defined variables [16]. Thus, unlike the propensity score matching method, the hdPS approach can deal with the difficulties of the high-dimensional framework of the health-care database by an automated algorithm for identifying variables across a wide range of available covariates. The hdPS was defined as the probability of experiencing an SRE on a set of 700 empirically selected confounding variables derived from seven variable dimensions: (1) inpatient diagnoses, (2) outpatient diagnoses, (3) drug prescriptions, (4) outpatient treatment, (5) inpatient treatment and surgeries, (6) aids, and (7) remedies. In addition, further covariates (age, sex, days of sick leave and Charlson Comorbidity Index) were entered in the hdPS model. Finally, a 1:1 nearest neighbor propensity score matching with maximum caliper 0.01 was applied.

### Outcomes and analyses

Baseline characteristics of patients were measured within the 12-month period prior to the index date (SRE event or randomly assigned index date) and were compared between the two patient groups, once before and once after matching. HCRU and cost outcomes were measured from the time of SRE or the randomly assigned index date (in case of non-SRE patients) until the end of the study period (30/06/2018), censoring for (i) death, (ii) initiation of a BTA-therapy, and (iii) later SRE (for the non-SRE group), whichever came first. To account for differences in the length of observational period, all outcomes were reported as means per patient-year (ppy), by dividing the total number of events (or incurred cost) by the total number of observational years within the observed group.

Rate ratios were calculated to estimate the influence of SREs on each HCRU and cost outcome. All health-related resources and incurred costs were accounted for in our analysis based on the following categorizations: outpatient physician visits, outpatient drug prescriptions, hospitalizations, inpatient rehabilitations, medical aids, and remedies. The total sum of all direct costs was then calculated. Moreover, costs have been reported for patients who were deceased during the follow-up period vs. patients who survived. Finally, a time-series analysis of the monthly costs within 2 years of an SRE event was performed.

Within the SRE group, outcomes were reported for subgroups as defined by (a) SRE type (by first observed event: pathological fracture, spinal cord compression, surgery to bone, radiation to bone, or various SREs at the same time) and (b) ST-type (latest documented ICD-10 code before or at time of index: malignant neoplasm of bronchus and lung (C34), malignant neoplasm of prostate (C61), malignant neoplasm of breast (C50), malignant neoplasm of kidney, except renal pelvis (C64), malignant neoplasm of the bladder (C67), or “other cancers”).

Our analysis reported percentages, mean [standard deviations (SD)] and median [interquartile range (IQR)]; *p* values for comparisons between datasets, were provided wherever applicable. Statistical comparisons were performed using Pearson’s chi-squared test, Mann–Whitney *U* test or *t* test, depending on the type and distribution of the variable.

The analysis was performed with Microsoft SQL Server 2008, Microsoft Excel 2010 (Microsoft Corporation, Redmond, WA, USA) and Stata version 14.1 software (Stata Statistical Software: Release 14. StataCorp, College Station, TX, USA). Data were analyzed descriptively. Percentages, mean [standard deviations (SD)] and median [interquartile range (IQR)], and *p* values for the comparisons between datasets, were provided where applicable.

## Results

### Study cohort and hdPS matching

**Fig. 1:**
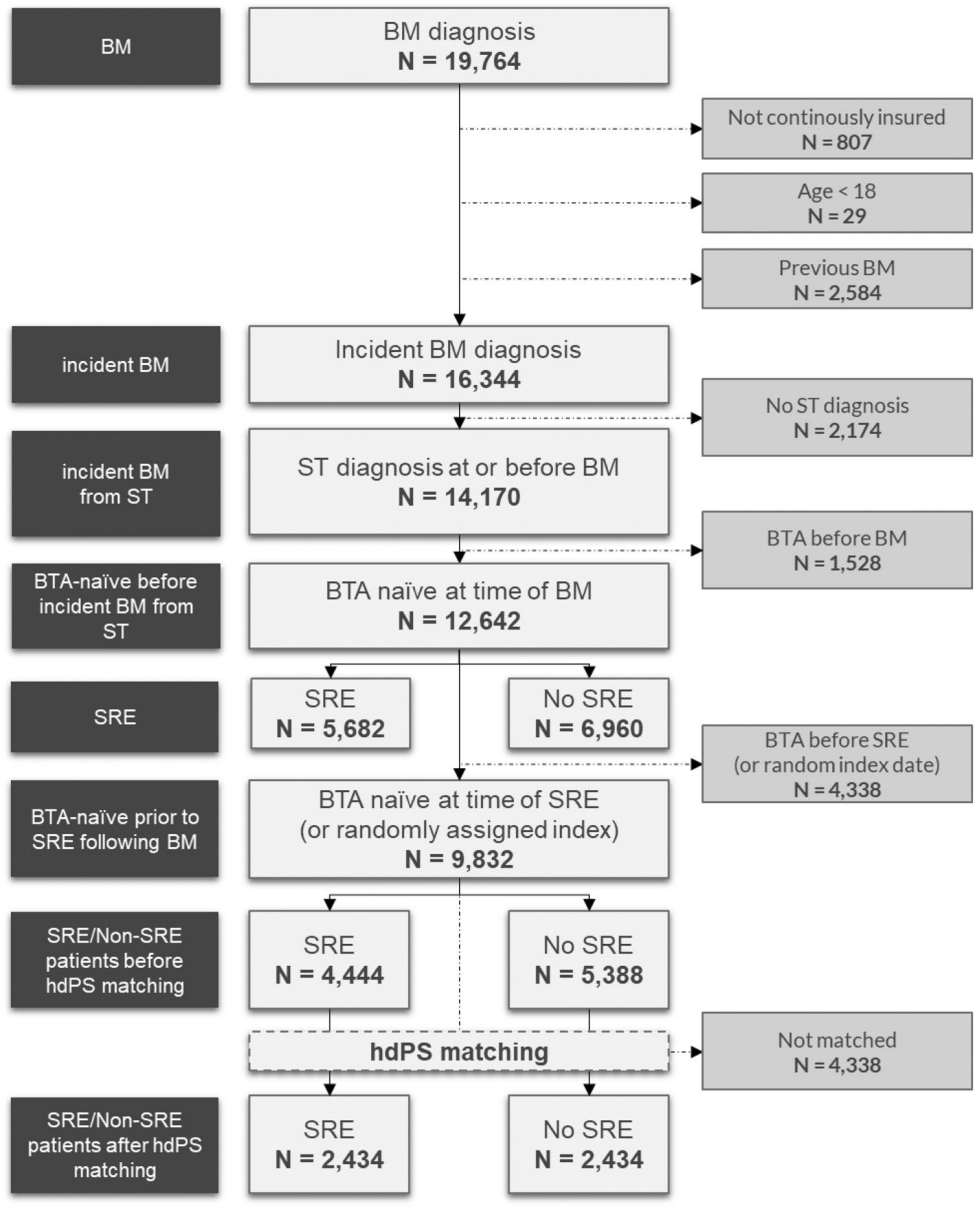
Patient attrition chart

**Fig. 2:**
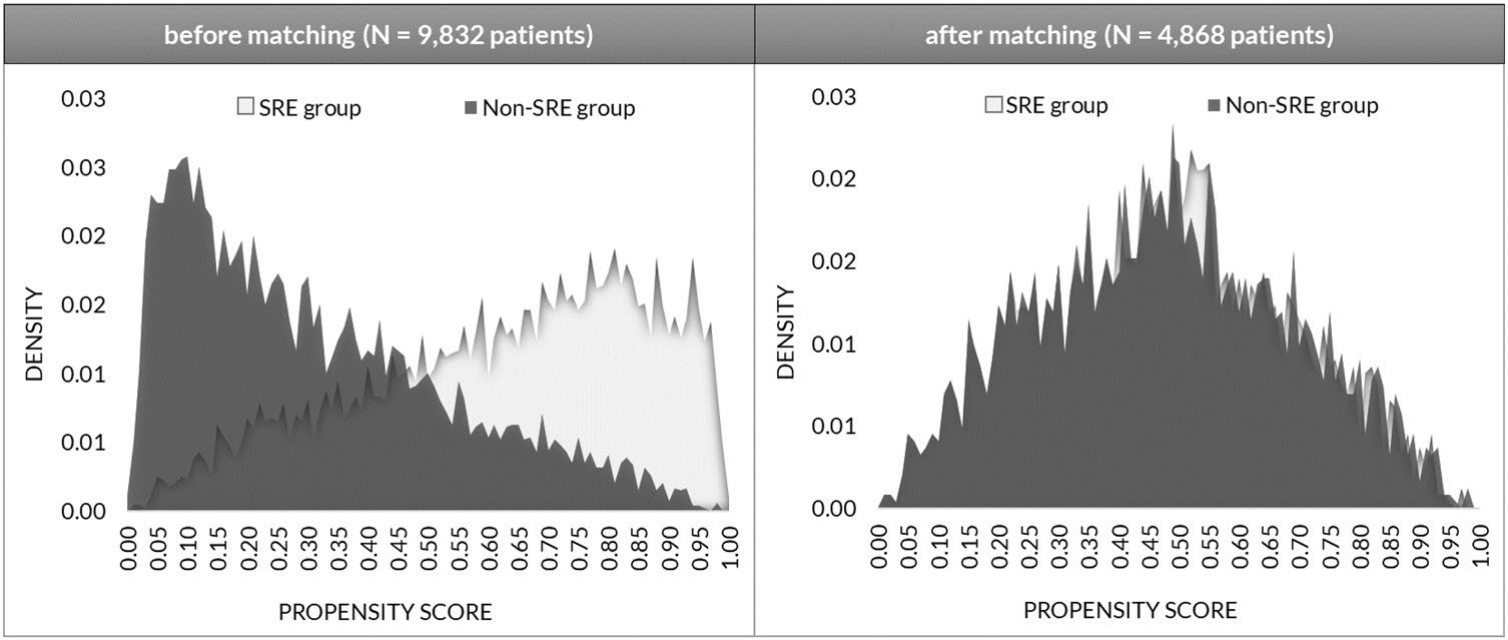
Propensity score distribution

The stepwise inclusion of patients for the initial cohort of incident BTA-naïve BM patients with/without an SRE event is described in Fig. 1. In total, 9,832 patients satisfied all inclusion criteria. Of those patients, 45.20% experienced at least one SRE (*n* = 4444) and 54.80% did not (*n* = 5388). After matching, 2,434 SRE and 2,434 non-SRE patients were included in the final cohorts. Table 1 provides an overview of patient characteristics for each cohort before and after matching. Figure 2 shows the propensity score distribution before and after matching.

**Table 1:** Baseline characteristics of matched and unmatched cohorts

Variable	Unmatched cohorts				Matched cohort			
	SRE (*N* = 4444)	Non-SRE (*N* = 5388)			SRE (*N* = 2434)	Non-SRE (*N* = 2,434)		
	Mean	Mean	Standardized difference	*p* value	Mean	Mean	Standardized difference	*p* value
Age at index	70.25	71.74	− 12.40	< 0.001	70.87	71.07	− 1.70	0.559
Proportion female patients	0.42	0.35	− 13.90	< 0.001	0.39	0.39	1.00	0.724
Charlson Comorbidity Index (CCI)	10.67	11.18	− 19.10	< 0.001	10.85	10.86	− 0.20	0.936
Hospitalizations	3.78	3.86	− 2.20	0.282	3.89	3.79	2.60	0.364
Outpatient GP visits	5.16	5.02	6.20	0.002	5.15	5.10	1.80	0.523
Days absent from work	2.59	2.71	− 0.60	0.758	2.76	2.62	0.80	0.777

### Baseline characteristics

Mean age at index date was 70.87/71.07 years in the SRE/non-SRE groups (*p* = 0.559), and 39.07%/38.58% were female (Table 2). The mean (median) Charlson Comorbidities Index (CCI), measured within the 12-month baseline period before the index date, was 10.85 (11) in SRE patients and 10.86 (11) in non-SRE patients. Within the group of matched SRE patients, 808 (33.20%) patients experienced a pathological fracture, 731 (30.03%) a spinal cord compression, 505 (20.75%) radiation to bone, 131 (5.38%) a bone surgery, and for 259 (10.64%) patients multiple SRE codes at the index day were documented. Concerning ST types, the majority of patients suffered from lung cancer (566, 23.25%), followed by prostate cancer (443, 18.20%), breast cancer (369, 15.16%), kidney cancer (159, 6.53%) and bladder cancer (95, 3.90%). A significant proportion of patients was identified with other or multiple cancer codes (802, 32.95%). Baseline characteristics for SRE subgroups are presented in Table 2 as well.

**Table 2:** Baseline characteristics of SRE subgroups

	Non-SRE group	SRE group	Within SRE group: SRE type				
			Pathological fracture	Spinal cord compression	Surgery to bone	Radiation to bone	Different SREs
	*N* = 2434	*N* = 2434	*N* = 808	*N* = 731	*N* = 131	*N* = 505	*N* = 259
Age; mean (SD) / Median	71.07 (12.19) / 73	70.87 (12.02) / 73	73.40 (11.64) / 76	71.41 (12.14) / 74	66.89 (11.51) / 70	66.69 (11.81) / 68	71.59 (10.88) / 73
Females;* N* (%)	939 (38.58)	951 (39.07)	346 (42.87)	276 (37.81)	46 (35.11)	170 (33.66)	113 (43.30)
CCI; mean (SD) / Median	10.86 (2.70) / 11	10.85 (2.65) / 11	10.99 (2.80) / 11	10.90 (2.70) / 11	10.09 (2.76) / 10	10.73 (2.80) / 10	10.94 (2.62) / 11

	Within SRE group: ST type					
	Lung cancer	Prostate cancer	Breast cancer	Kidney cancer	Bladder cancer	Other cancers
	*N* = 566	*N* = 443	*N* = 369	*N* = 159	*N* = 95	*N* = 802
Age; mean (SD) / Median	67.46 (10.96) / 68	77.59 (8.70) / 79	71.22 (12.14) / 74	72.22 (10.98) / 74	70.38 (12.41) / 73	69.18 (12.89) / 72
Females;* N* (%)	164 (28.98)	0 (0.00)	366 (99.19)	68 (42.77)	20 (21.05)	333 (41.52)
CCI; mean (SD) / Median	10.81 (2.32) / 10	11.43 (2.68) / 11	10.34 (3.09) / 10	11.62 (2.69) / 12	11.26 (2.63) / 11	10.60 (2.55) / 10

Characteristics of patients within non-SRE and SRE-group, as well as ST/SRE subgroups of the SRE cohort. Variables were observed within 12 months prior to the SRE index date (or randomly assigned index)

*CCI* Charlson Comorbidity Index; *SD* standard deviation; *SRE* skeletal-related event; *ST* solid tumor

On average, patients were followed for 288.46 days in the SRE group (median 99 days) and 154.92 days in the non-SRE group (median 38 days). Censoring happened at either (i) BTA therapy initiation, (ii) death, (iii) an observed SRE event (only for non-SRE patients), or (iv) 30/06/2018 (end of the observation window). The minority of patients was observed until the end of data availability (10.81% of SRE patients; 8.18% of non-SRE patients). Censoring due to initiation of BTA therapy after SRE happened more often in the SRE cohort (27.73% of all patients) compared to the non-SRE group (12.70%). Death was observed in 61.46% of SRE patients and 76.38% of non-SRE patients, taking above censoring into account. Finally, 2.79% of non-SRE patients were censored due to an SRE after the inclusion period.

### HCRU and cost

Table 3 summarizes the observed HCRU and cost comparison between SRE and non-SRE patients. Inpatient costs accounted for 59.60% of the overall cost within the SRE group, and 52.87% within the non-SRE group, and thereby hospitalizations were the main cost driver. SRE patients experienced 35.80 hospitalization days ppy, compared to 30.80 days within the non-SRE group (*p* < 0.001). This resulted in higher direct cost ppy for all-cause hospitalizations, with 14,199.27€ for SRE patients versus 10,787.31€ for non-SRE patients (*p* < 0.001).

**Table 3:** HCRU and cost ppy for SRE and non-SRE patients

	SRE patients	Non-SRE patients	Rate ratios (RR) (*p* value)
HCRU			
Number of all-cause hospitalizations, ppy	3.64	3.33	1.09 (< 0.001)
Number of SRE-related hospitalizations, ppy	0.48	0.00	–
Number of all-cause hospitalization days, ppy	35.80	30.80	1.16 (< 0.001)
Number of SRE-related hospitalization days, ppy	7.74	0.00	–
Number of outpatient GP visits, ppy	4.99	5.00	1.00 (> 0.100)
Number of outpatient specialist visits, ppy	12.70	12.07	1.05 (< 0.001)
Number of rehabilitation stays, ppy	1.38	0.25	5.55 (< 0.001)
Number of outpatient drug prescriptions, ppy	43.19	42.89	1.01 (< 0.001)
Cost [€]			
Hospitalizations, ppy	14,119.27	10,787.31	1.31 (< 0.001)
Outpatient visits, ppy	1,637.50	1,498.75	1.01 (< 0.001)
Rehabilitations, ppy	213.37	38.07	5.61 (< 0.001)
Outpatient prescriptions, ppy	6,126.75	6,771.99	0.90 (< 0.001)
Aids and Remedies, ppy	1,592.66	1,307.15	1.22 (< 0.001)
Total cost, ppy	23,689.54	20,403.27	1.16 (< 0.001)

The number of events and related costs (in €) following the SRE index date (or randomly assigned index) in consideration of the follow-up time, compared between SRE patients and non-SRE patients based on rate ratios

*HCRU* health care resource use; *GP* general practitioner; *ppy* per patient year; *RR* rate ratios; *SRE* skeletal-related event

SRE patients experienced on average 12.70 outpatient specialist visits ppy, and thus 1.05 times as many visits as non-SRE patients (12.07; *p* < 0.001). For general practitioner (GP) visits, no significant difference was found between both groups. Overall direct costs associated with outpatient visits was 1,637.50€ in the SRE group compared to 1,498.75€ in the non-SRE group (*p* < 0.001).

Cost for outpatient prescriptions was higher in the non-SRE group (6,771.99€), compared to the SRE group (6,126.75€; *p* < 0.001). Main drivers in that respect were a higher number of prescriptions of analgesics (pyrazolones), opioids and proton inhibitors in the SRE versus non-SRE group (see Supplementary Fig. 1).

**Fig. 3:**
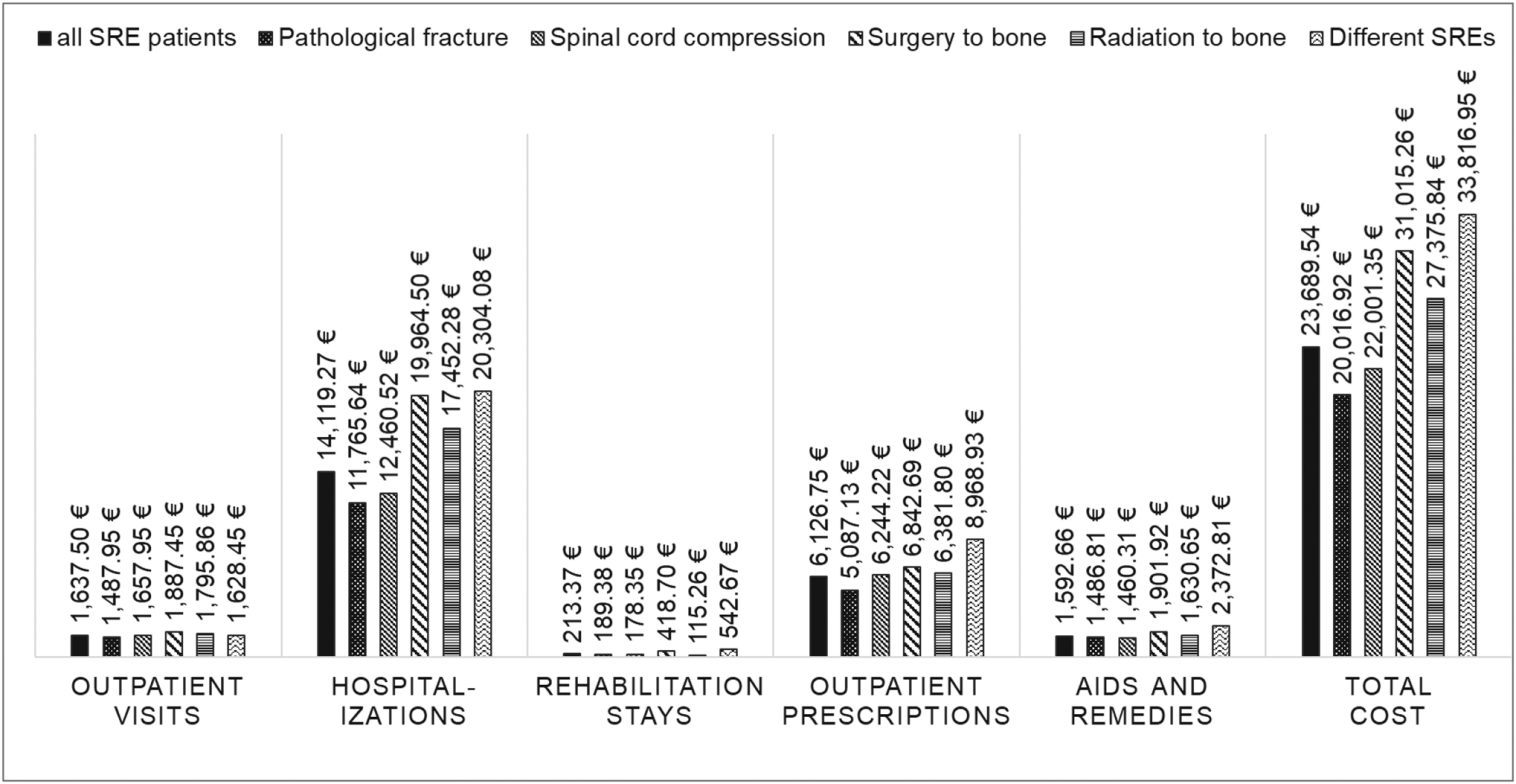
Cost by SRE type. Cost (in €) following the SRE index date, reported ppy for different SRE types as subgroups of the SRE patient cohort. ppy, per patient year; SRE, skeletal-related event

**Fig. 4:**
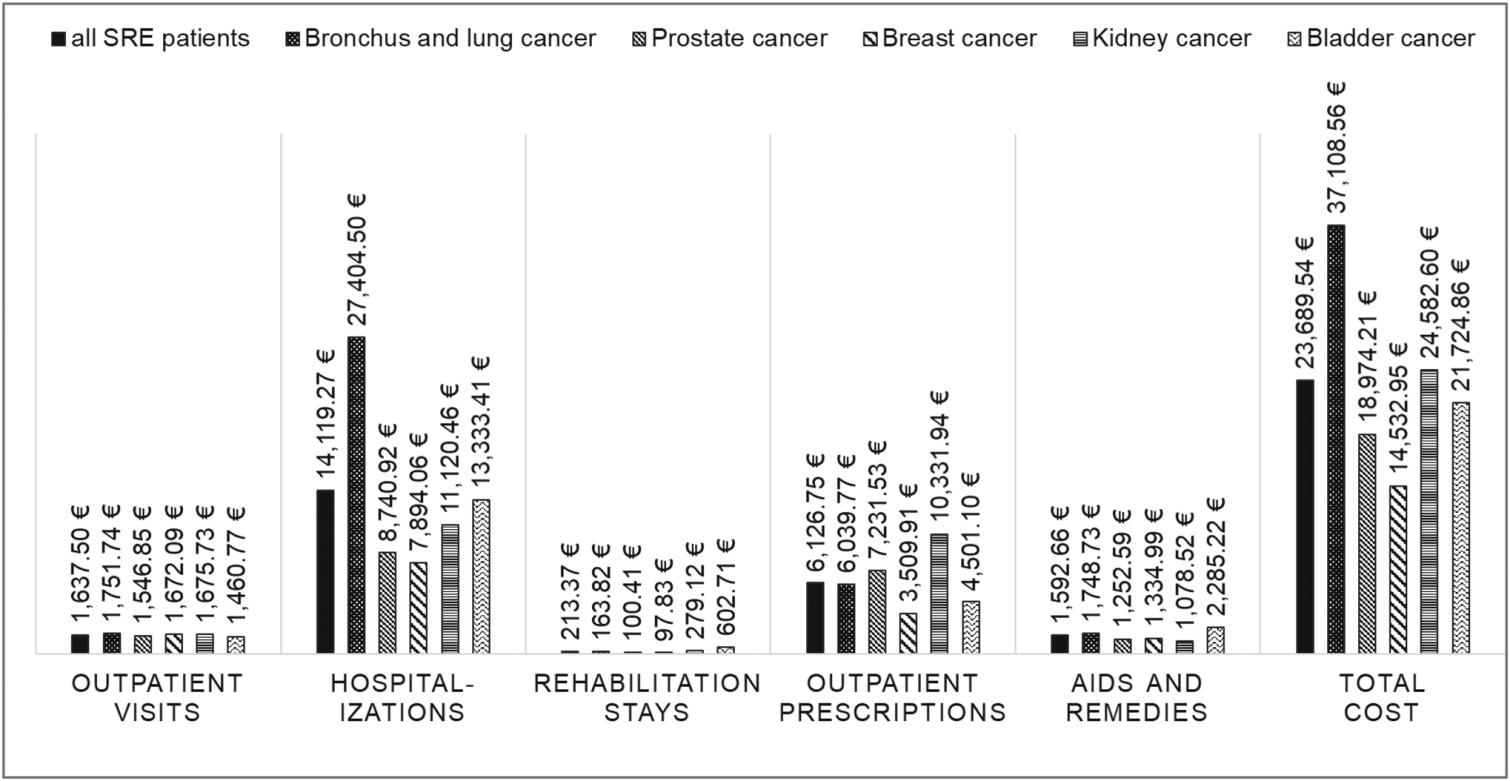
Cost by ST type. Cost (in €) following the SRE index date, reported ppy for different ST types as subgroups of the SRE patient cohort. ppy, per patient year; SRE, skeletal-related event

Based on the SRE patients only, subgroup-specific HCRU is shown in Table 4 and direct costs are presented in Figs. 3 and 4. SRE patients with multiple SRE types at the index date had the highest total direct healthcare costs (33,816.95€), followed by patients with surgery to bone (31,015.26€ ppy). Lowest overall costs were incurred by patients with pathological fracture (20,016.92€ ppy). This group also had a relatively low number of hospitalization days (30.71 ppy). Among ST types, lung cancer patients had the highest direct healthcare costs (37,108.56€ ppy), with significantly more hospitalization days (66.99 ppy) and higher costs for inpatient stays (27,404.50€ ppy) compared to the remaining patients.

**Table 4:** HCRU for SRE and ST subtypes

	SRE type				
	Pathological fracture	Spinal cord compression	Surgery to bone	Radiation to bone	Different SREs
	*N* = 808	*N* = 731	*N* = 131	*N* = 505	*N* = 259
HCRU					
Number of all-cause hospitalizations, ppy	3.09	3.42	3.73	4.67	4.80
Number of SRE-related hospitalizations, ppy	0.51	0.37	0.61	0.47	0.75
Number of all-cause hospitalization days, ppy	30.71	29.54	38.90	50.58	53.42
Number of SRE-related hospitalization days, ppy	7.74	7.85	6.25	11.85	6.98
Number of outpatient GP visits, ppy	4.97	4.93	5.01	5.11	5.19
Number of outpatient specialist visits, ppy	11.95	14.34	11.42	11.73	11.12
Number of outpatient drug prescriptions, ppy	41.50	43.75	41.14	41.66	53.16

	ST type					
	Lung cancer	Prostate cancer	Breast cancer	Kidney cancer	Bladder cancer	Other cancers
	*N* = 566	*N* = 443	*N* = 369	*N* = 159	*N* = 95	*N* = 802
HCRU						
Number of all-cause hospitalizations, ppy	7.22	2.44	2.34	2.34	3.26	4.22
Number of SRE-related hospitalizations, ppy	0.85	0.33	0.31	0.48	0.47	0.53
Number of all-cause hospitalization days, ppy	66.99	20.14	36.48	22.75	31.23	36.66
Number of SRE-related hospitalization days, ppy	13.13	5.42	5.26	8.65	7.22	8.61
Number of outpatient GP visits, ppy	5.48	4.83	4.60	4.89	4.71	5.23
Number of outpatient specialist visits, ppy	9.98	13.96	14.54	13.42	12.53	11.58
Number of outpatient drug prescriptions, ppy	55.22	37.90	36.85	42.34	43.38	46.26

The number of events and related cost (in €) following the SRE index date reported ppy, for different SRE and ST types as subgroups of the SRE patient cohort

*HCRU* health care resource use; *GP* general practitioner; *ppy* per patient year; *RR* rate ratios; *SRE* skeletal-related event; *ST* solid tumor

### Comparing costs among deceased and non-deceased patients

**Fig. 5:**
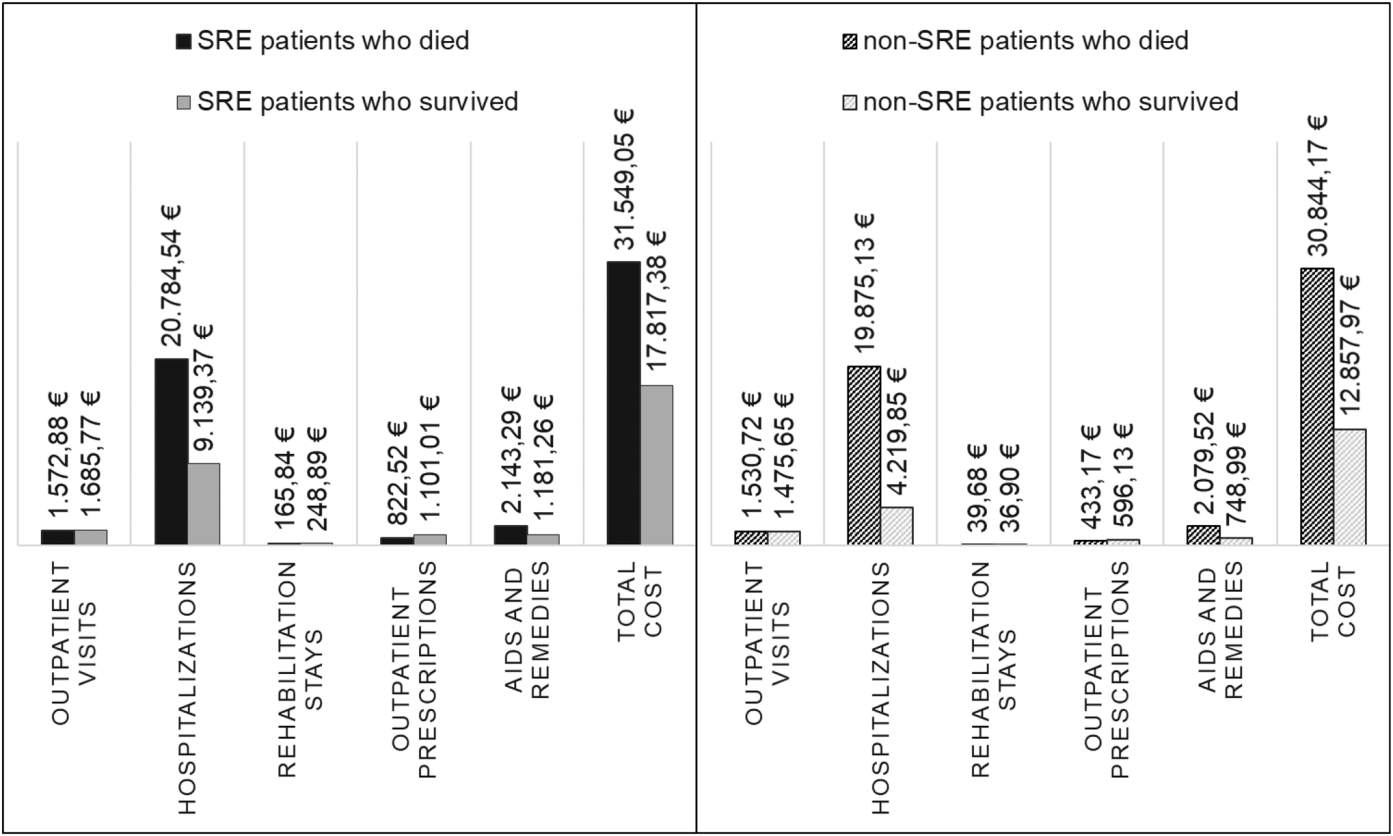
Cost among deceased and non-deceased patients. Cost (in €) following the index date, reported ppy for patients who were deceased during the follow-up period versus patients who survived. ppy, per patient year; SRE, skeletal-related event

A subgroup analysis of healthcare costs in deceased and non-deceased patients was performed for both SRE and non-SRE matched cohorts. Figure 5 illustrates differences in cost among patients who died and those who were alive at the end of observation. Ultimately, total costs were substantially higher for patients who died during the observation period (SRE cohort: 31,549.05€/non-SRE cohort: 30,844.17€) than for those who survived (17,817.38€/12,857.97€). When controlling for survival status between cohorts, average costs were higher in the SRE cohort than those in the non-SRE cohort across all expense categories.

### Cost development over time

**Fig. 6:**
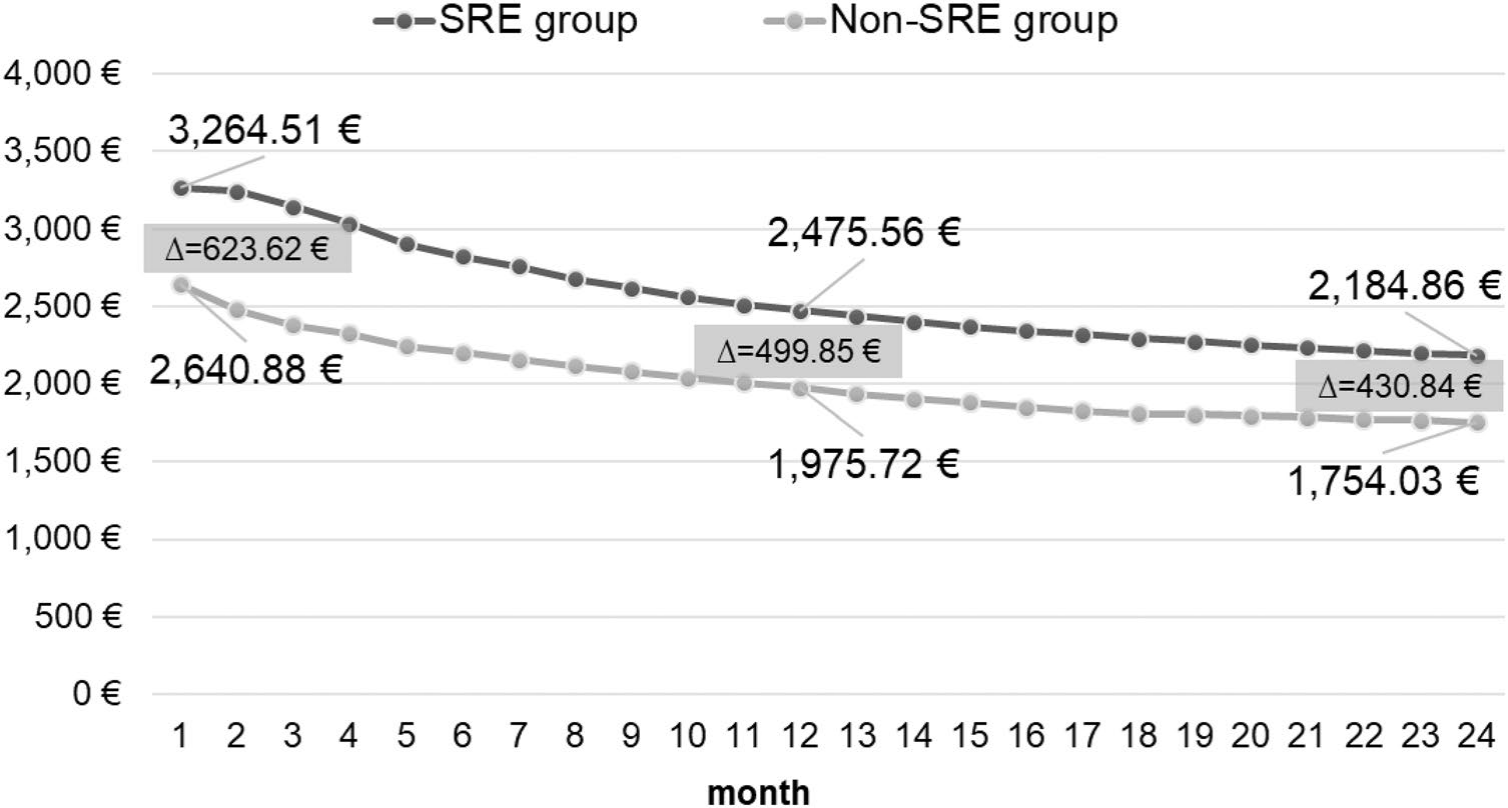
Cost development (per patient-month) over the first 24 months (cumulative). Time-series analysis of incurred cost (in €) in each month (reported per patient month) within the first two years after SRE event (or respective random index date) compared between SRE and non-SRE patients. SRE, skeletal-related event

We analyzed direct overall healthcare cost development within the initial 24 months following the index date and compared costs between SRE and non-SRE patients. Total costs per patient-month were higher for SRE versus non-SRE patients from the start of the observation (Fig. 6). However, the crude cost difference of 623.62€ per patient-month in the beginning reduces to 499.85€ after 12 months, which translates into a cost rate ratio of 1.25 (*p* < 0.001). After 24 months, average total costs per patient-month were still higher in the SRE group, but the cost difference decreased further to 430.84€ with a stable cost ratio of 1.25.

## Discussion

To investigate the economic consequences of undertreating or delaying treatment of cancer patients, our study assessed the incremental burden of SREs within a cohort of BTA-naïve patients with bone metastases from solid tumors, utilizing an hdPS methodology to improve comparability of patient cohorts. The analysis was based on a large number of patients (3.2 million insured persons, > 3% of the overall German population) with comparably long availability of data (early 2010-mid 2018). Thus, we were able to apply strict inclusion criteria in line with our study objectives and nevertheless could observe a population of interest with a reasonable sample size. Therefore, we interpret our results to be generalizable for Germany, even if the sickness fund providing the data (AOK PLUS) is only active in the German states Saxony/Thuringia. Due to uniform healthcare regulations throughout Germany, the treatment of AOK PLUS patients is not expected to differ from other regions. Previous studies in other disease areas have confirmed this [17].

Our study identified a total of 12,642 patients with BM from ST, who were naïve to BTA therapy at the time of their BM diagnosis. Among those, 44.95% of patients experienced at least one SRE during the observation period. Comparable estimates of 38.7–55% [18–24] have been reported in previous analyses of patients with BM, although these studies also included BTA-treated patients. Additionally, it has been shown that the proportion of BM patients experiencing SREs is higher in those patients who received therapy with bisphosphonates, explaining our slightly lower number of reported SRE cases [18]. One similar US-based study which also observed BTA-naïve BM patients, reported that 59.4% of patients with an incident BM diagnosis experienced an SRE within 48 months after their initial diagnosis (with 46.3% of SREs occurring within 12 months) [25].

In our study, 10.78% (i.e. 1,528 patients) of 14,170 total patients with an incident BM from ST received a BTA therapy before their incident BM diagnosis. Among the remaining 12,642 patients, 34.31% (i.e. 4,338 patients) started a BTA therapy after the incident BM diagnosis before the occurrence of the first SRE (or random index date in the non-SRE group). Finally, 20.21% (i.e. 984 patients) of 4,868 matched patients started a BTA therapy only after the occurrence of an SRE. Thus, the number of BTA-untreated patients at the time of incident BM diagnosis was as high as 59%, even though the guidelines recommend a BTA therapy [2, 7]. This is also higher than previously reported [26–28]. In any event, BTA undertreatment of patients poses a relevant challenge in the real-world treatment of BM patients. Further research is needed to understand the underlying reasons for this.

The strengths of our analysis are the unbiased nature of the dataset and use of a hdPS methodology with a high degree of comparability of our patient cohorts. Finally, 2,434 SRE and 2,434 non-SRE patients have been included in our comparison of HCRU and cost of these patients. Although the propensity score distribution shows the quality of the conducted matching, we cannot exclude a possible indication bias related to unknown confounders. Moreover, as the analysis focused on BTA-naïve patients at the date of the incident SRE event, the random index assignment for the non-SRE cohort was done before the hdPS matching.

We observed substantial differences both in the follow-up time and the mortality between the patient cohorts. There may be different reasons for this. One reason could be the random assignment of index dates for non-SRE patients, that took place before the hdPSM was performed. This was done as the baseline characteristics (12 months before the index date) were needed for the matching itself and because of the fact that patients with previous BTA therapy were excluded from the analysis. Thus, the distribution of index dates might have been different between SRE and non-SRE patients after the matching. Additionally, in the non-SRE patients, a substantially higher percentage of patients was censored due to initiation of BTA therapy. Consequently, follow-up periods were shorter in that period and death could be observed less often in SRE patients. Generally, there was an observed divergence in follow-up times, due to different proportions of event censorship between cohorts. To account for these differences, outcomes were observed ppy.

Moreover, SREs were partly defined by treatments including radiotherapy rather than the diagnosis of events, and specific SREs (e.g. fractures) are more likely to occur in patients with fewer mobility constraints. We conclude that the performance status of some non-SRE patients might have been too poor to initiate treatment, leading to a higher representation of seriously-ill patients in the non-SRE group. Thus, our analysis may underestimate incremental costs of SREs, as more end-of-life patients were assigned to the non-SRE group, which is also expressed in the form of a substantial higher mortality risk in the non-SRE group observed in our study, despite use of the hdPSM methodology.

Although data from statutory health insurance funds offer a comprehensive basis to respond to underlying study objectives, a number of limitations may arise by the absence of precise clinical data [29]. The identification of SREs and STs was only based on available inpatient and outpatient diagnosis and procedure codes, which were validated by a clinical expert treating the target patients during the study design phase. Nevertheless, we acknowledge a potential risk of incorrect assignment of patients to the observed groups, as no clinical data for confirmation of diagnoses and procedures were available.

Generally, data on outpatient care are collected for the purpose of accounting, which is conducted on a quarterly basis in Germany. As a result of this and the nature of the corresponding dataset, it is not possible to observe whether a patient visited the same doctor more than once in a given quarter. Conversely, visits to different doctors in the same quarter are reported separately. Thus, the lack of available data on the number of times a patient visits each of his/her doctors per quarter represents one limitation of the applied methodology.

We identified a considerable economic burden associated with SREs in our target population. SREs are associated with a higher total direct healthcare cost. This is also the case when comparing costs incurred by SRE patients and non-SRE patients who died during observation. One of the main reasons for this is a higher burden resulting from inpatient treatment. Our findings show that the number of hospitalization days is about 1.16 times higher in the SRE group than in the non-SRE group. Also, SRE patients have significantly more inpatient rehabilitation stays than non-SRE patients (RR: 5.55) and visited outpatient specialists more frequently (RR: 1.05). Previous European investigations have mainly focused on the evaluation of HCRU and cost of SREs in all patients with BM from ST, regardless of their treatment [30, 31]. To our knowledge, no comparable data on BTA-naïve patients exist for Germany and thus, this is the first study to show the impact of SREs in a population of BTA-naïve patients. A comparable study in a population of BTA-naïve BM patients was conducted based on US Market Scan Data in the US [25]. It reported a higher HCRU per year in SRE patients too, attributing its findings to observed differences in inpatient admissions (3.3 versus 1.5) and ER visits (6.6 versus 3.8). Also, the mean length of stay in the SRE group was significantly longer compared to the non-SRE counterparty (15.7 versus 8.3 days) in the US study [25]. However, since the US and German health-care systems strongly differ from each other [32], a direct comparison of our results and those of the US study is challenging.

We found that the total direct healthcare cost ppy was 23,690€ in the SRE group and 20,403€ in the non-SRE group. Therefore, the total health-care cost ppy for SRE patients was 1.16 times as high as the cost for non-SRE patients. In the above-mentioned US Market Scan Data analysis, the total cost was 168,277$ ppy in SRE patients and 101,020$ in non-SRE patients [25]. Another US study, based on SEER data, found considerable higher cost for SRE patients, compared to patients without SREs [33]. Due to the specifics of the US and German markets, the absolute costs are very different from each other. However, the proportion of inpatient costs as a percentage of all direct costs was similar between our investigation (59.60%/52.87% of the total cost in SRE/non-SRE patients) and the US analysis (63% of the incurred costs) [25].

## Conclusion

In response to the general evidence gap, this study uniquely contributes to understandings of incremental HCRU and cost of BTA-naive BM patients due to an SRE event in Germany. We found a considerable economic burden associated with SREs. Moreover, we identified a high percentage of BTA-untreated patients in the BM cohort (around 59%). Consequently, our study emphasizes the importance of a guideline-adherent BTA treatment not only from the clinical perspective but also from a health-economic perspective.

## Supplementary Information

Below is the link to the electronic supplementary material.

Supplementary file1 (PDF 95 KB)

Supplementary file2 (DOCX 16 KB)

Supplementary file3 (DOCX 14 KB)

## Data Availability

The data that support the findings of this study are abstracted from individual patient records. Data were available for research purposes from the sickness fund upon request, in an anonymized form. Due to restrictions around revealing patients’ confidential information, data were used under license for the current study, and so are neither publicly available nor can be shared further.

## Code availability

Non-applicable.
