# Supplementary figures and images for: Healthcare resource utilization and associated cost of patients with bone metastases from solid tumors who are naïve to bone-targeting agents: a comparative analysis of patients with and without skeletal-related events
Source: Eur J Health Econ. 2021 Jan 18;22(2):243–54. doi: 10.1007/s10198-020-01247-z (PMC7881971; doi:10.1007/s10198-020-01247-z)

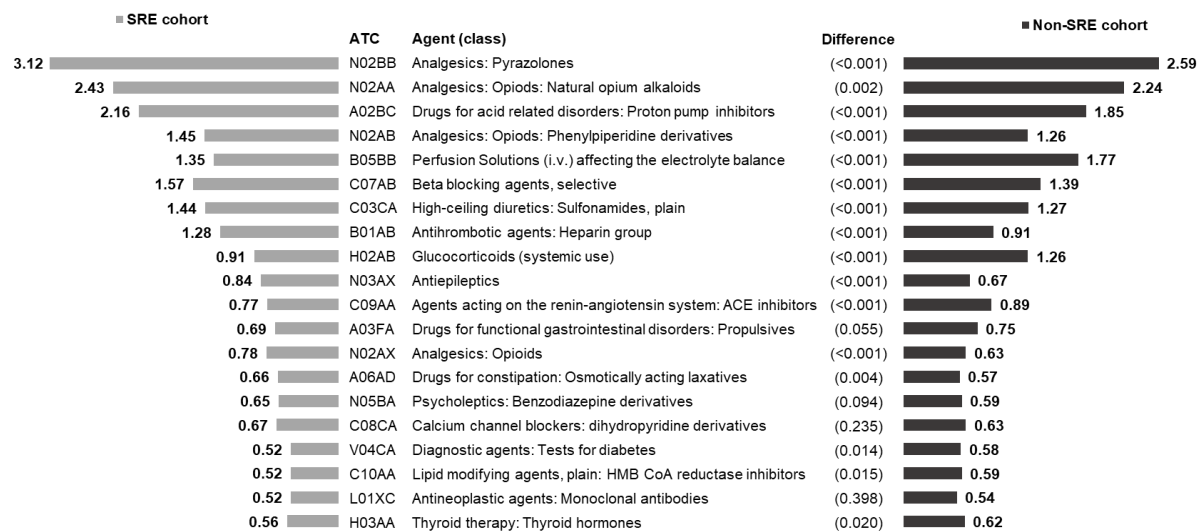

Supplement: Supplementary file 1 — Supplementary file1 (PDF 95 KB) [file 10198_2020_1247_MOESM1_ESM.pdf]
