# Supplementary material for: Healthcare resource utilization and associated cost of patients with bone metastases from solid tumors who are naïve to bone-targeting agents: a comparative analysis of patients with and without skeletal-related events
Source: Eur J Health Econ. 2021 Jan 18;22(2):243–54. doi: 10.1007/s10198-020-01247-z (PMC7881971; doi:10.1007/s10198-020-01247-z)
Supplement: Supplementary file 2 — Supplementary file2 (DOCX 16 KB) [file 10198_2020_1247_MOESM2_ESM.docx]

**Supplementary Table 1: List of Skeletal-Related Events**

| **CODE** | **DIAGNOSIS OR PROCEDURE** |
| --- | --- |
| **Pathological fractures** | |
| M48.5 (ICD-10) | Collapsed vertebra, not elsewhere classified |
| M84.4 (ICD-10) | Pathological fracture, not elsewhere classified |
| M90.7 (ICD-10) | Fracture of bone in neoplastic disease |
| S02 (ICD-10) | Fracture of skull and facial bones |
| S12 (ICD-10) | Fracture of neck |
| S22 (ICD-10) | Fracture of rib(s), sternum and thoracic spine |
| S32 (ICD-10) | Fracture of lumbar spine and pelvis |
| S42 (ICD-10) | Fracture of shoulder and upper arm |
| S52 (ICD-10) | Fracture of forearm |
| S62 (ICD-10) | Fracture at wrist and hand level |
| S72 (ICD-10) | Fracture of femur |
| S82 (ICD-10) | Fracture of lower leg, including ankle |
| S92 (ICD-10) | Fracture of foot, except ankle |
| T10 (ICD-10) | Fracture of upper limb, level unspecified |
| T12 (ICD-10) | Fracture of lower limb, level unspecified |
| **Spinal Cord Compression** | |
| G95.2 (ICD-10) | Cord compression, unspecified |
| G95.8 (ICD-10) | Other specified diseases of spinal cord |
| G95.9 (ICD-10) | Disease of spinal cord, unspecified |
| G99.2 (ICD-10) | Myelopathy in diseases classified elsewhere |
| M43.9 (ICD-10) | Deforming dorsopathy, unspecified, Curvature of spine NOS |
| M47.0 (ICD-10) | Anterior spinal and vertebral artery compression syndromes |
| M47.1 (ICD-10) | Other spondylosis with myelopathy |
| M48.5 (ICD-10) | Collapsed vertebra, not elsewhere classified |
| M50.0 (ICD-10) | Cervical disc disorder with myelopathy |
| M50.1 (ICD-10) | Cervical disc disorder with radiculopathy |
| M50.9 (ICD-10) | Cervical disc disorder, unspecified |
| M51.0 (ICD-10) | Lumbar and other intervertebral disc disorders with myelopathy |
| M51.1 (ICD-10) | Lumbar and other intervertebral disc disorders with radiculopathy |
| M54.5 (ICD-10) | Low back pain |
| M54.6 (ICD-10) | Pain in thoracic spine |
| M54.9 (ICD-10) | Dorsalgia, unspecified |
| T09.3 (ICD-10) | Injury of spinal cord, level unspecified |
| **Surgery to Bone** | |
| 5-76 (OPS) | Surgery for facial skull fractures |
| 5-77 (OPS) | Other surgeries on facial skull bones |
| 5-78 (OPS) | Surgery on other bones |
| 5-79 (OPS) | Reduction of fracture and dislocation |
| 5-81 (OPS) | Arthroscopic joint surgery |
| 5-83 (OPS) | Surgery on the spine |
| **Radiation to bone** | |
| Z51.0 (ICD-10) | Radiotherapy session |
| 8-520 (OPS) | Surface radiation therapy |
| 8-521 (OPS) | Ortho-voltage radiotherapy |
| 8-522 (OPS) | High-voltage radiotherapy |
| 8-523 (OPS) | Other high-voltage radiotherapy |
| 8-524 (OPS) | Brachytherapy with enclosed radionuclides |
| 8-525 (OPS) | Other brachytherapy with enclosed radionuclides |
| 8-526 (OPS) | Radioactive moulages |
| 8-527 (OPS) | Design and adaptation of fixation and treatment aids in radiotherapy |
| 8-528 (OPS) | Irradiation simulation for external radiation and brachytherapy |
| 8-529 (OPS) | Irradiation planning for percutaneous radiation and brachytherapy |
| 8-52a (OPS) | Proton therapy |
| 8-52b (OPS) | Carbon ion therapy |
| 8-52c (OPS) | Other heavy ion therapy |
| 8-52d (OPS) | Intraoperative radiotherapy with X-rays |
| 8-530.1 (OPS) | Therapy with open radionuclides in bone metastases |

***Note:*** *OPS (Operationen- und Prozedurenschlüssel) is a coding system used in Germany to characterize and encode a range of operations, procedures, and general medical measures.*
