# Supplementary material for: Healthcare resource utilization and associated cost of patients with bone metastases from solid tumors who are naïve to bone-targeting agents: a comparative analysis of patients with and without skeletal-related events
Source: Eur J Health Econ. 2021 Jan 18;22(2):243–54. doi: 10.1007/s10198-020-01247-z (PMC7881971; doi:10.1007/s10198-020-01247-z)
Supplement: Supplementary file 3 — Supplementary file3 (DOCX 14 KB) [file 10198_2020_1247_MOESM3_ESM.docx]

**Supplementary Table 2: List of Identified Bone Targeting Agents**

| **ATC Code** | **AGENT** | |
| --- | --- | --- |
| M05BX04 | Denosumab | |
| M05BA01 | Etidronic acid | *Bisphosphonates* |
| M05BA02 | Clodronic acid |  |
| M05BA03 | Pamidronic acid |  |
| M05BA04 | Alendronic acid |  |
| M05BA05 | Tiludronic acid |  |
| M05BA06 | Ibandronic acid |  |
| M05BA07 | Risedronic acid |  |
| M05BA08 | Zoledronic acid |  |
| M05BB01 | Etidronic acid and calcium, sequential | *Bisphosphonates, combinations* |
| M05BB02 | Risedronic acid and calcium, sequential |  |
| M05BB03 | Alendronic acid and colecalciferol |  |
| M05BB04 | Risedronic acid, calcium and colecalciferol, sequential |  |
| M05BB05 | Alendronic acid, calcium and colecalciferol, sequential |  |
| M05BB06 | Alendronic acid and alfacalcidol, sequential |  |
| M05BB07 | [Risedronic acid and cholecalciferol](https://www.whocc.no/atc_ddd_index/?code=M05BB07&showdescription=yes) |  |
| M05BB08 | Zoledronic acid, calcium and colecalciferol, sequential |  |
| M05BB09 | Ibandronic acid and colecalciferol |  |
